# Investigating the paternal genetic structure and migration history of Chinese Qiang minority by Y-chromosome short tandem repeats

**DOI:** 10.1098/rsos.242046

**Published:** 2025-05-21

**Authors:** Guang-Yao Fan, Ying Zhu, En-Na Wang, Tian-Ge Ju

**Affiliations:** ^1^ School of Medicine, Shaoxing University, Shaoxing, Zhejiang, People’s Republic of China; ^2^ School of Life and Environmental Sciences, Shaoxing University, Shaoxing, Zhejiang, People’s Republic of China

**Keywords:** Y-chromosomal short tandem repeats, patrilineal history, genetic structure, Qiang minority, machine learning

## Abstract

Known for its legendary history and peculiar culture, Chinese Qiang minority aroused interest among geneticists and anthropologists. However, the paternal genetic landscape of its sub-branches coupled with its migration history has never been uncovered. In this study, 37 Y-chromosomal short tandem repeat (Y-STR) loci of three Qiang ethnic groups (*N* = 564) were investigated to shed light on their paternal genetic landscape. The phylogenetic reconstruction among 29 populations was conducted based on their Y-chromosomal haplotypes. The genetic affinities of the four different Qiang subgroups exhibited obviously variant when compared with Han, Yi or Tibetan in Tibetan-Yi corridor. Based on machine learning method, the predicted Y chromosome haplogroups demonstrated the predominance of O2a1 and O2a2. The haplogroup distributions were compared among 40 contemporary ethnic groups in West China and DNA samples of 214 ancient humans from 59 archaeological sites. The results supported that Wenchuan Qiang had historical links with the ancients in West Liao River (WLR) region. Moreover, intrapopulation gene flow was analysed using Migrate-n. Bidirectional migration was proved to be the most frequent model among the Qiangic populations while the unidirectional migration was only observed from Wenchuan to Li County.

## 1. Introduction

Qiang ethnic minority mainly inhabited a mountainous region in the middle section of Tibetan-Yi corridor [1,2]. They are supposed to be one of the ancestors of Sino-Tibetan populations that can be traced back to about 6000 years ago [3,4]. The earliest inscriptions of text ‘Qiang’ on oracle bones emerged 3000 years earlier. However, the characteristic varies to some extent between the modern Qiang people and the ancient ‘Qiang’ people [1]. The former one mainly refers to those non-Han people living in the upper Min River Valley and Beichuan area such as the counties of Mao, Wenchuan, Li, Heishui and Songpan. Nonetheless, Qiangic languages include at least 10 sub-branches, and there is a possibility that Qiang people migrated from all directions. Figuring out the genetic characteristics of the paternal lineage will provide significant clues to the origin of modern Qiang, hierarchical structure and historical migration.

Due to the uniparental inheritance mode, Y-chromosomal short tandem repeats (STRs) and single nucleotide polymorphism (SNPs) are powerful tools to infer the paternal genealogy, human origin and migration trajectories [5]. As suggested by Y-chromosomal evidence, the modern Qiang people might originate from the Sino-Tibetan populations [6,7]. Y-SNP loci possess much lower mutation rates than Y-STR loci [8] and perform an important role in studying more remote evolutionary history [9] and demographic processes [10] by comparing of the Y chromosome haplogroups. Benefiting from advance machine learning techniques, Y-chromosomal haplogroups can be predicted by Y-STR haplotypes [11–13]. Such prediction was based on the correlation between Y-chromosomal haplogroup and corresponding haplotypes [14,15]. Therefore, exceedingly laborious and time-consuming Y-SNP genotyping may become unnecessary.

Up to now, the interpopulation genetic relationships and migration routes in Tibetan-Yi corridor, the core settlement of Qiang, is still controversial. In this study, the investigated Y-STRs had shed light on the population stratification, historical gene flow and phylogenetic relationships among the Qiang minority and other Chinese ethnic groups in Tibetan-Yi corridor. Meanwhile, the study will also contribute to the regional-effective forensic reference database.

## 2. Material and methods

### 2.1. Sample preparation

A total of 564 healthy Qiang males were recruited for this study. All the participants were unrelated and belonged to three different counties, Li (*n* = 159), Mao (*n* = 72) and Wenchuan (*n* = 333) of Ngawa Tibetan and Qiang Autonomous Prefecture (electronic supplementary material, figure S1). The household registration certificates were identified to assure their national attribute. We confirmed that all the subjects were born and had been resident in their hometown for at least three generations. Blood spots were collected on fast technology for analysis (FTA) cards (Whatman, Clifton, NJ, USA). This study was carried out in line with the Declaration of Helsinki [16]. Each volunteer participant read and signed an informed consent form. Additionally, Y-STR genotyping data from the other populations living in West China were also collected for comparison (electronic supplementary material, table S1). Furthermore, Y-chromosomal haplogroup frequencies were acquired not only from the contemporary populations living in West China (electronic supplementary material, table S2), but also from the ancient DNA belonged to the widespread archaeological sites in China (electronic supplementary material, table S3).

### 2.2. Polymerase chain reaction amplification and genotyping

One punch generated from each of the FTA cards was placed straight into the 25 μl polymerase chain reaction (PCR) system. Fluorescent multiplex amplification was performed on a GeneAmp^®^ PCR System 9700 (Thermo Fisher Scientific, USA) using a commercial Y-STR kit of AGCU Y37 Kit (AGCU ScienTech Incorporation, Wuxi, Jiangsu, China) [17].

PCR amplicons of 37 loci and 5 Y-InDel markers PCR products were detected using the ABI 3500 Genetic Analyzer (Applied Biosystems, USA). GeneMapper^®^ ID-X (Applied Biosystems, USA) was applied for allele allocation. All the genotyping experiments strictly followed the manufacturer’s instruction. Laboratory internal control standards and kit controls were used for quality control. The proficiency of our lab was certified through the participation in the Y-STR typing quality test as organized by YHRD (https://yhrd.org). The Y-STR haplotypes included in this study have been submitted to the YHRD and assigned the accession numbers YA005114. Our laboratory has been accredited by the China National Accreditation Service for Conformity Assessment (CNAS).

### 2.3. Statistical analyses

Allele and haplotype frequencies of 37 Y-STRs were estimated through counting. Single-marker genetic diversity (GD) and haplotype diversity (HD) were calculated using the *Nei*’s formula [18]. The discrimination capacity (DC) was computed as a ratio of the number of unique haplotypes to the overall number of haplotypes. Based on 15 shared Y-STR loci, the online analysis of molecular variance (AMOVA) calculation tool of YHRD (https://yhrd.org/pages/tools/amova) was used for measuring *R*_ST_ genetic distances among 29 populations in West China (electronic supplementary material, table S1). Furthermore, the neighbour-joining (NJ) phylogenetic tree was reconstructed with the PHYLIP v. 3.6.95 [19] and visualized using Evolview v. 3 [19]. Besides, a well-established machine learning (ML) technique for classification, linear discriminant analysis (LDA), was utilized for predicting categories [20]. To evaluate the preliminary performance of Y-STR haplotypes in classifying diverse populations in Tibetan-Yi corridor, genotyping data from Han, Qiang, Tibetan and Yi populations were adopted in LDA (electronic supplementary material, table S1) via the R package ‘lda’ based on 23 single-copy Y-STR loci.

As a non-parametric supervised learning method, the k-nearest neighbour (kNN) was proved to be a powerful tool both for regression and classification [21]. For example, it had been used for allocating Y-chromosomal haplogroups based on the haplotypes [11,12,22]. In order to build a kNN predictor dedicated to the Qiang minority, pairwise Y-chromosomal haplotype and corresponding haplogroup from previous study [23] had participated in the calculation via the R package ‘knn’ [24]. There were 23 single-copy Y-STR loci involved in the training and testing datasets. Subsequently, the performance of the developed kNN predictor were revealed by a series of statistics. Moreover, the software of Network 10.2. was utilized to generate cladogram for the predominate haplogroups based on Median-joining model [25], while the multi-copy loci were excluded ahead. In addition, the multidimensional scaling (MDS) plot was conducted via the IBM SPSS^®^ software [26,27] based on the haplogroup frequency dataset of 40 contemporary populations (electronic supplementary material, table S2) and two ancient human clusters from West Liao River (WLR) basins and Hengbei with relatively adequate samples (electronic supplementary material, table S2). Principal component analyses (PCA) were also carried out by using the Origin version 2018 [28,29] based on the same dataset.

Migration rates were inferred among the three studied groups and Beichuan Qiang by Migrate v. 4.4.3. This analysis was based on the coalescence theory and used Bayesian inference to estimate posterior probability densities of all the parameters of a Qiangic population model [30]. The sampling increment and burn-in values were set to 1000 and 5000, respectively. Metropolis–Hastings sampling was used in the procedure to evaluate the parameters [31,32]. Four ‘hotter’ chains at different temperatures (1.0, 1.5, 3.0 and 10 000) were adopted to probe more genealogy space than the ‘cold’ chains [33,34].

## 3. Results

### 3.1. Haplotype diversity

Haplotype data for 37 Y-STR loci of Qiang ethnic minority in this study are listed in electronic supplementary material, table S2. Null allele was detected once in DYS448, as confirmed by experimental replication (electronic supplementary material, table S4). In 564 Qiang males, a total of 563 haplotypes were observed, 562 of which were single-copy (electronic supplementary material, table S4). Overall, the observed HD of 37 Y-STR reached 0.9835, and DC reached 0.9982. The most frequent Y-STR haplotypes were found twice. Electronic supplementary material, table S5 lists the allelic frequencies and gene diversity values. In addition to the three multi-copy loci of DYS385, DYF387S1 and DYS527, 26 out of 32 single-copy loci showed a greater GD value than 0.5 with an exception of four loci: DYS645, DYS391, DYS437 and DYS438 (electronic supplementary material, figure S2).

### 3.2. Meta-population comparisons

**Figure 1.**
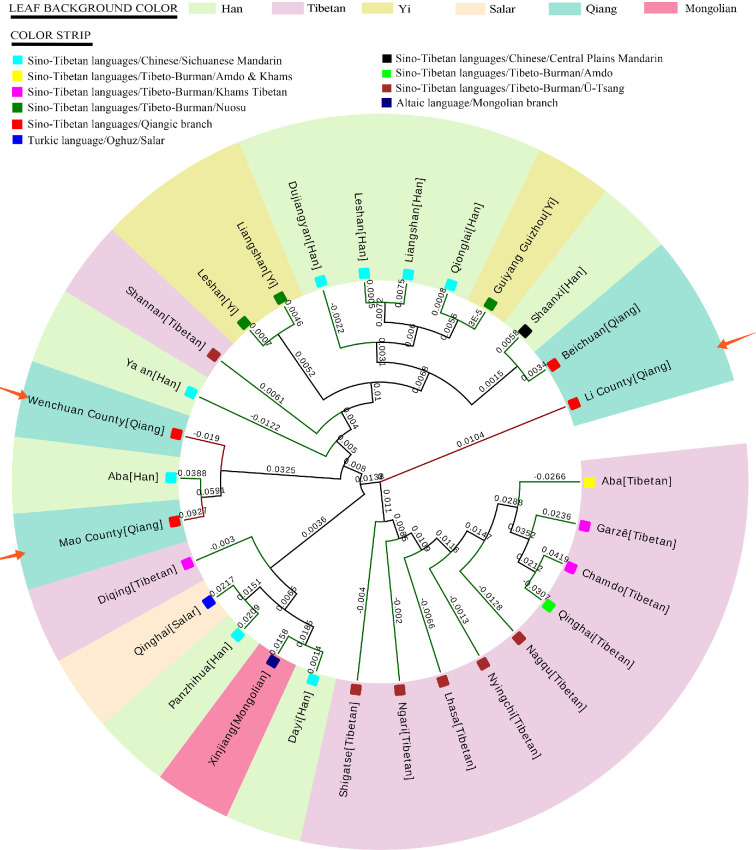
Dendrogram of 29 populations constructed on *R*_ST_ genetic distances. The three Qiang ethnic groups sampled from the counties of Li (*n* = 159), Mao (*n* = 72) and Wenchuan (*n* = 333) in Ngawa Tibetan and Qiang Autonomous Prefecture are indicated by orange arrows.

The pairwise genetic distances (*R*_ST_) between the three Qiang ethnic groups and the reference populations were obtained. As shown in electronic supplementary material, table S6, the lowest *R*_ST_ value was observed between the Han populations from Panzhihua and Dayi (−0.0419), while the highest value was found between Chamdo Tibetan and Liangshan Han (0.3427). Among all the non-Qiangic populations, Ya'an Han and Chamdo Tibetan were found to be the closest (−0.0274) group and the farthest (0.3292) one to the Qingic populations, respectively. Generally, the Han, Tibetan and Yi populations in West China were more closely related genetically with Qiang from the counties of Beichuan, Li and Wenchuan than the Qiang from Mao County. Meanwhile, the NJ phylogenetic tree was contributed (figure 1). Beichuan Qiang people shared the same clade with Shaanxi Han at the far end of the phylogenetic tree. Conversely, the Qiang people from Li County were located near the root of the phylogenetic tree. In addition, Aba Han had a closer genetic link to the Qiangic populations from the counties of Wenchuan and Mao. Some of the Tibetan inhabitants (Diqing and Shannan) residing at the most southern edge of Tibetan-Yi corridor fell outside the macro-clade of Tibetan people.

### 3.3. Machine learning-based linear discriminant analysis

**Figure 2.**
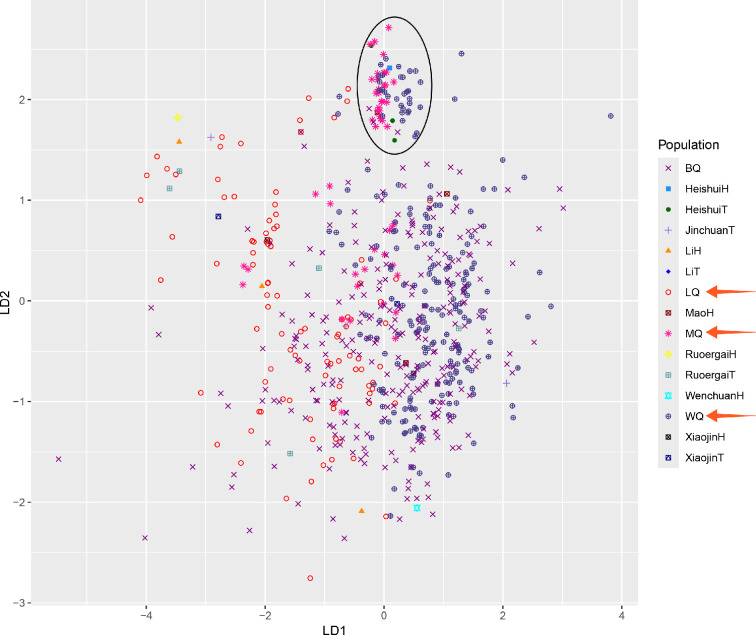
** **Population substructure reconstruction for the males in Ngawa Tibetan and Qiang Autonomous Prefecture based on 23 single-copy Y-STR loci in LDA. All samples with null alleles were excluded for analysis. The three Qiang ethnic groups (LQ, MQ and WQ) sampling from the counties of Li (*n* = 159), Mao (*n* = 72) and Wenchuan (*n* = 333) are indicated by orange arrows in the legend. The abbreviations of H and T at the end of each population’s name stand for Han and Tibetan nationalities, respectively.

Based on 23 single-copy Y-STR loci, LDA was implemented among the 16 populations (Qiang, Han, Tibetan and Yi) derived from the reference database (electronic supplementary material, table S1). The trend of gathering was observed among the four nationalities. Yet, lots of Qiang individuals from Li County and Tibetan individuals from Garzê scattered on the right of the plot (electronic supplementary material, figure S3A). Such trend was still notably when only Qiangs and Tibetans were retained for analysis (electronic supplementary material, figure S3B). Besides, LDA was also implemented among Han and Qiangic groups. It was visible that Qiangic populations from the counties of Wenchuan and Li can be separated from each other by an artificial dividing line, but other populations cannot (electronic supplementary material, figure S3C). Meanwhile, another dividing line also existed between the two Qiangic populations from the counties of Wenchuan and Li in consideration of the Yi and Qiang individuals (electronic supplementary material, figure S3D). Furthermore, when only the Qiang minority was considered in LDA, the three studied Qiangic populations mainly distributed in different ellipses while Beichuan Qiang were in dispersed distribution (electronic supplementary material, figure S3E). In order to predict categories more finely in Ngawa Tibetan and Qiang Autonomous Prefecture, all our subgroups in Aba were involved in LDA. Figure 2 shows that lots of Qiang individuals from the counties of Mao and Wenchuan gathered with Tibetan and Han from Heishui County.

### 3.4. Haplogroup prediction and network analysis

**Figure 3.**
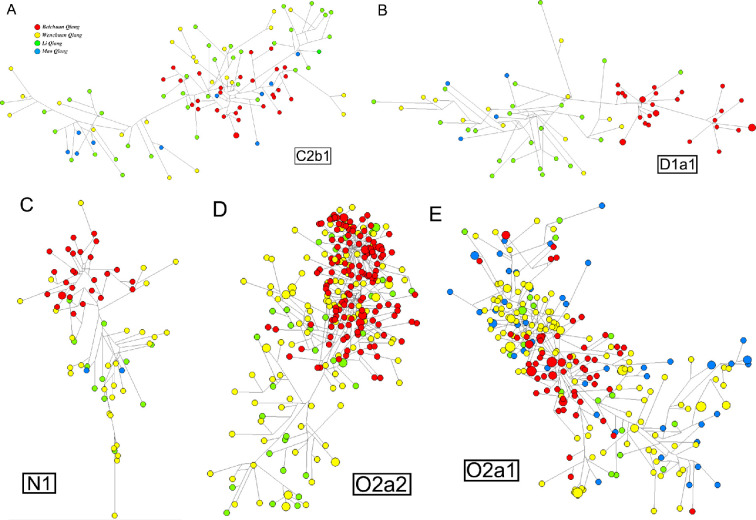
Median-joining networks of the five dominant Y-haplogroups C2b1, D1a1, N1, O2a2 and O2a1 for all the four Qiangic populations based on 23 Y-STR loci. The published Beichuan Qiang and three studied Qiangic populations from the counties of Li, Mao and Wenchuan were included during the network constructions. The area of the circles is directly proportional to the frequency of haplotypes, while the lines connecting them are proportional to the number of mutational steps.

Moreover, the kNN algorithm was utilized to build a robust predictor based on training dataset for the Qiangic ethnic groups. Subsequently, the performance of the predictor was evaluated for allocating eight unique haplogroups (C2b1, D1a1, N1, O1a1, O1b1, O2a1, O2a2 and Q) based on the testing dataset (overall average accuracy: 96.17%). The satisfactory confusion matrix shows the counts of each haplogroup that was correctly or incorrectly predicted (electronic supplementary material, table S7). The overall statistics indicated that the predictor for most of the haplogroups possessed high sensitivity and specificity (electronic supplementary material, table S8). The kNN predictor had been shown to be robust compared with the Whit Athey’s Haplogroup Predictor tool (https://www.hprg.com/hapest5/) based on Bayesian probability (electronic supplementary material, table S9). The distribution of Y-chromosomal haplogroup frequencies was illustrated in electronic supplementary material, figure S4, and the first two dominant haplogroups were O2a1 (34.75%) and O2a2 (26.06%) in the three studied Qiangic populations. These findings were consistent with the former report, which suggested Beichuan Qiang possessed high percentages of haplogroup O2 and O1 [23]. Among the 40 present-day populations in West China, the proportions of the C2 were varied (electronic supplementary material, figure S5). The higher proportion of the C2b1 haplogroup was found in Qiang and Han in West China compared with Yi and Tibetan. Notably, the highest proportion of the C2 haplogroup was observed in the Li Qiang. Furthermore, the median-joining Y-STR networks for the dominant haplogroups of C2b1, D1a1, N1, O1b1, O2a2 and O2a1 were constructed by the software of Network 10.2., respectively (figure 3). The most prominent feature of all the networks was the high concentration of Beichuan Qiang. In order to reveal the similarity or heterogeneity of the Y-chromosomal haplogroup constituent between the Qiang minority and other populations living in West China, Y-SNPs data from 37 non-Qiangic ethnic groups (electronic supplementary material, table S2) were involved in comparison. As shown in electronic supplementary material, figure S5, no notable similarity was found between the three studied Qiangic populations and non-Qiangic groups. But the obvious similarity was observed between Beichuan Qiang (BCQ) and Sichuan Han (SCH). Generally, the haplogroup constituent of Qiang minority was more similar to the Han in West China.

### 3.5. Estimation of the migration routes for Qiangic minority

**Figure 4.**
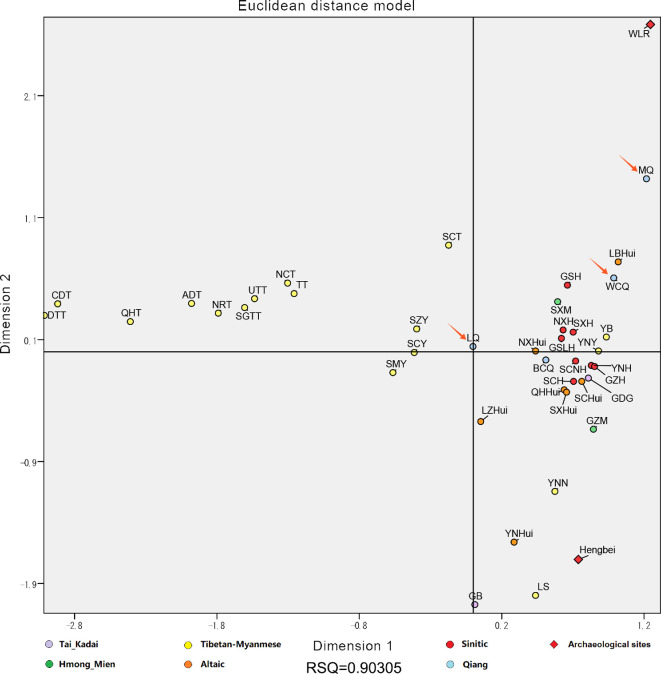
Multidimensional scaling analysis among 40 ethnic groups in West China and ancient people from the Hengbei and the WLR (West Liao River) region based on Y-SNP haplogroup frequencies. The three Qiang ethnic groups (LQ, MQ and WCQ) sampled from the counties of Li (*n* = 159), Mao (*n* = 72), and Wenchuan (*n* = 333) are indicated by orange arrows in the chart.

**Figure 5.**
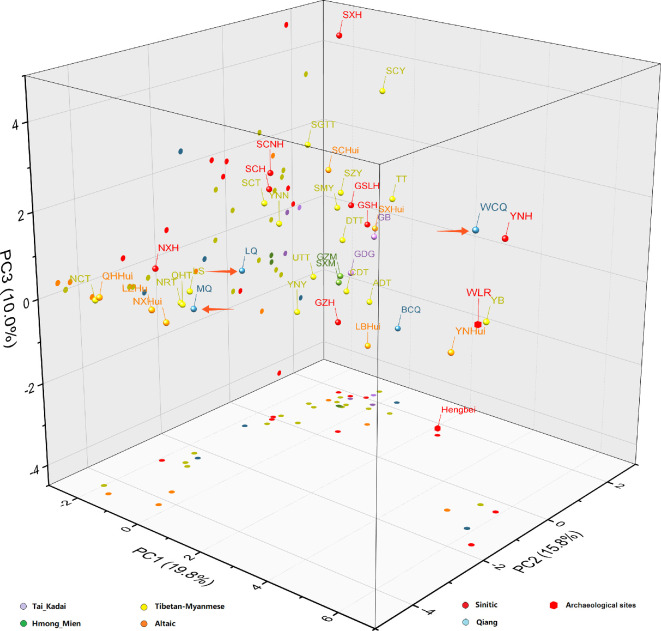
Three-dimensional principal components analysis (PCA) demonstrated the genetic differences and similarities among 40 ethnic groups in West China and ancient people from the Hengbei and the WLR region based on Y-SNP haplogroup frequencies. The three Qiang ethnic groups (LQ, MQ and WCQ) sampling from the counties of Li (*n* = 159), Mao (*n* = 72) and Wenchuan (*n* = 333) are indicated by orange arrows in the chart.

Ancient DNA can be used to reveal genetic connections between modern Qiang and ancient people that will benefit to reconstruct the migration route and direction for Qiang minority. Cultural archaeological areas were basically sorted by latitude and longitude, followed by presumed dates. The ancient human remains that belonged to the haplogroups of O2a2, O2a1, O1b1, D1, N, Q1a1 and C2b1 are shown in grey, while O2 and O2a are indicated in light grey (electronic supplementary material, table S3). Geographic locations of the archaeological sites (D1, N, Q1a1 and C2b1) are illustrated in electronic supplementary material, figure S1. The sites of Hengbei exhibited similar Y-chromosomal haplogroup constitution (O2a, O2a2, O1b1 and Q1a1) with modern Qiang. The prevalence of Q1a1 haplogroups in Hengbei was remarkable. Meanwhile, the Neolithic sites of Taosi_Longshan and XiaoWu_YangShao geographically close to Hengbei, and their Q1a1a1 and O2 were also observed in Modern Qiang. Nevertheless, they encompassed a large temporal range and were limited by a very small sample size. Thus, the data from Longshan and YangShao cannot be used for analysing the migration of Qiang. Meanwhile, the WLR ancients in northeast China, which exhibited similar Y-chromosomal haplogroup constitution (O2, O2a2, N and C2b1) with modern Qiang, were mainly composed of Dashanqian Dadianzi and Halahaigou. It was remarkable that the proportion of N haplogroups was quite high in these archaeological sites. The above trends were further illustrated in the MDS plot (figure 4). Nearly all the Tibetan and Yi populations were widely scattered in the left part, other ethnic groups were mainly concentrated in the right part. The Qiang from Mao County and Wenchuan were close to the WLR ancients, while Mao Qiang was relatively far from the cluster. Moreover, variances totalling 45.6% were extracted from the overall genetic variability by the first three components (PC1: 19.8%; PC2: 15.8%; PC3: 10.0%) in the three-dimensional PCA plot (figure 5). Generally, no significant clustering trend was observed. The Qiangic populations from the counties of Mao and Li were relatively close to each other. Besides, the Qiangic population from Wenchuan was close to the WLR ancients.

Alternatively, relatively large number of Y-STR haplotype data from contemporary populations have distinct advantage in studying migration route and direction. The intrapopulation gene flow among the Qiangic populations was investigated in this study. At the first level, gene flow was evaluated between adjacent Qiangic populations under the models 1−3 (electronic supplementary material, figure S6). Meanwhile, the models 4−7 were designed for investigating the population movements between the non-adjacent Qiangic populations in consideration of the divergence and continuing gene flow. The results indicated that bidirectional model (model 1) was the most probable migration routes in the pairwise counties of Beichuan-Mao, Mao-Wenchuan and Mao-Li (electronic supplementary material, table S10). Meanwhile, the model 2 had the highest log marginal likelihood between Wenchuan and Li which indicated a unidirectional migration of Qiang from Wenchuan to Li. Secondly, at the level two (models 4−7 in electronic supplementary material, figure S6), the model 7 from Beichuan to Li via Mao County, was proved to be the most probable migration model among all non-adjacent pairs (electronic supplementary material, table S11). It indicated that Li splits off Beichuan, and Mao simultaneously splits off Beichuan and Li. Therefore, Qiang’s complex movements along Tibetan-Yi corridor could be revealed and illustrated in electronic supplementary material, figure S7.

## 4. Discussion

### 4.1. Genetic affinities

A high HD value was observed in the cluster composed of the three studied Qiangic populations. Based on the *R*_ST_ genetic distance, Ya'an Han was found to be the closest (−0.0274) one to the Qingic populations among all the non-Qiangic populations. The negative value may be due to the limited sampling pool of Ya'an Han (*n* = 9). However, it is obviously that Han groups (except Dayi and Panzhihua) were more genetically close to Wenchuan Qiang than the other populations. Meanwhile, the genetic affinities among Qiang, Han, Tibetan and Yi hints a conspicuous assimilation within these geographic close populations. This finding is consistent with our previous study which had exhibited the genetic homogeneity between Yi and Beichuan Qiang [2]. This observation was confirmed in the dendrogram where the Qiangic groups fell into a big cluster composed of many Yi and Han groups (figure 1). On the dendrogram Beichuan Qiang people shared the same clade with Shaanxi Han at the far end of the phylogenetic tree, which hints at a gene flow between these geographically close populations. Moreover, one branch of Qiang (Li County) was located near the root of the phylogenetic tree. It was consistent with the linguistic evidence of Qiangic languages, which was supposed to be the origin of all other Sino-Tibetan languages [3]. Moreover, genetic connections between early Di-Qiang and Han Chinese had been revealed by previous study via ancient DNA [35,36]. The Wenchuan Qiang and Mao Qiang have obvious genetic affinity to each other and combined a relatively independent branch on the dendrogram with Aba Han rather than Yi, Tibetan or the other Qiang groups. Nearly all the Tibetan groups fell into a relatively separate large branch except Shannan and Diqing. According to historical records, the Qiang area became a contested territory between Tang and Tubo in Tang Dynasty. The Qiang people had undergone many wars and were forced to accept some Tibetan cultures. But learning from our results, Qiang and Tibetan did not experience significant gene exchange, and basically retained their national patrilineal genetic traits. As an exceptional case, Shannan Tibetan and Li Qiang is worth for deeper investigation in the future. Since the *R*_ST_ genetic distances were measured based on limited 15 shared Y-STR loci, the intrapopulation heterogeneity among Qiang, Tibetan and Yi still needs more validation.

In order to finely depicture the relationship of the populations in Ngawa Tibetan and Qiang Autonomous Prefecture, all our subgroups in Ngawa were involved in LDA based on 23 Y-STR loci (figure 2), and Qiang males from the counties of Mao and Wenchuan gathering with Tibetan and Han from Heishui County. Since the population of the Qiang ethnic group in Heishui is much smaller than that in Maowen region, sampling the Qiang ethnic group in Heishui County is relatively difficult. However, it is not difficult to infer from the LDA results and geographical particularity that an important ethnic corridor along the Min River from Heishui to Wenchuan most likely had an important impact on the genetic structure of Maowen region. Someone believed that the Qiang minority originally lived on the prairie and had later undergone natural disasters and wars-driven southward migration. These early migrants were distributed along Min River and some areas of Peijiang River, including Songpan, Mao, Wenchuan, Li and Heishui County [23]. They had been active along Min River during the Han Dynasty based on the literature [3]. This history is generally consistent with our LDA findings. To linguists, both the Heishui Tibetans and the Maoweng Qiang speak the Qiang language of the Tibeto-Burman Qiang branch with slight differences in dialect. However, the Heishui people consider themselves Tibetans and have no connection to the Qiang people in the east. Although lots of Qiang individuals from the counties of Mao and Wenchuan gathering with Tibetan and Han from Heishui County in LDA, and similar ethnologic cultural traditions and legends can also be confirmed, such analysis has certain limitations given the insufficient sample size, and further investigation with an adequate sample size is needed in the future. While the investigation is not limited to Ngawa, some of the Li Qiang individuals cluster with Garzê Tibetan (electronic supplementary material, figure S6A,B). This could be a consequence of the gene exchange between them. Such ethnic fusion may also have led to the divergence between Li Qiang and Wenchuan Qiang in electronic supplementary material, figure S6C,D.

### 4.2. Haplogroups distribution

The higher frequent haplogroups of O2a1 and O2a2 is impressive, when the three studied Qiangic groups were considered as a whole (electronic supplementary material, figure S4). Compared with former study for Beichuan Qiang [23], haplogroup J and R were absent in the three studied populations. Meanwhile, the particularity of Beichuan Qiang was also illustrated in the networks. Besides, it was remarkable that O2a2 haplogroup was absent in Mao Qiang (figure 3). However, what kinds of fact caused the disappearance of the O2a2 branch people is still lacking of clues. The higher proportion of the C2b1 haplogroup was found in Qiang and Han in West China. As the typical haplogroup in the residents of the Eurasian temperate steppe, C2 was mainly found in northern Han populations and archaeological sites across vast continent covering seven millennia [34,37–39]. Comparison of the archaeological sites (electronic supplementary material, table S3), only the Qilangshan, Yikeshu, Zhukaigou, LongTouShan and Mogushan (C2b1), was geographically close to Central Plain which almost approached the extreme south of C2-derived lineages [40]. One of explanation for the higher C2b1 proportion in Qiang is that the Qiang in Ngawa accepted C2b1 immigrants from the north, and the immigrants integrated with them. It is worth exploring why the southbound migration has a more profound impact on the Qiang people instead of the Tibetan and Yi people. Alternatively, the Qiang C2a1 may itself be from the north as the Qiang’s main body, which obviously contradicts the previous linguistic conclusion that all Sino-Tibetan populations might have originated in western Sichuan [1].

Archaeological evidence suggested that the Yangshao Culture (approx. 7000 yr. BP) had its origin in the region of the Qiang people [36,41]. As shown in electronic supplementary material, figure S4, the archaeological sites of Yangshao Culture (XiaoWu and WangGou) located in Central Plain possessed the haplogroup of Q1a1 and O1b1, respectively. Among the four Qiangic groups, the Q haplogroup were only exited in the counties of Beichuan and Li, while the O1b1 was only absent in Mao Qiang (electronic supplementary material, figure S5). It hints some connection between Qiang (Beichuan and Li) and Yangshao Culture ancients or later Hengbei ancient.

The archaeological sites in WLR region including Dashanqian Dadianzi and Halahaigou exhibited much higher proportion of N haplogroups than the other sites. Surprisingly, they possessed O2, O2a2, N and C2b1 at once; those were the main haplogroups in modern Qiang. the number of ancient human samples available at each archaeological site is very limited, and we cannot use this to make substantiated predictions about the paternal genetic structure of that time. The results of PCA and MDS indicated that Wenchuan Qiang had historical links with the WLR ancients.

As the main body of Qiang minority, the distribution of O2 haplogroup is important to inference the present-day Qiang’s origin and its relationship with ancient Qiang. Combining historical and geographic information, the word ‘Qiang’ first appeared in the oracle bone inscriptions of the Shang Dynasty without clear meaning until Han Dynasty. Ancient Qiang were the natives of the He-Huang region, and they were forced to move westward by Huaxia. Some of them formed the Qiang people in He-Huang region and southwest region of Han Dynasty [42]. The archaeological sites of Taojiazhai, Lajia, Dacaozi, JinChanKou and Mogou all possessed O2 haplogroup and fell into the He-Huang region, which gave a support for Qiang’s southward migration. Therefore, a reasonable inference is that the O2 haplogroup of the contemporary Qiang people should be the real descendants of the ancient Qiang people in He-Huang area. Meanwhile, it is believed that one branch of the early migrants moved towards southwest and formed the Tubo, who are the ancestors of modern Tibetans, or other southwestern minorities through integrating with the local population [43,44]. Notably, there is not D haplogroup among the archaeological sites except Qinghai-Tibet Plateau (electronic supplementary material, table S3 and figure S5), while the proportion of D1 haplogroups was quite high in the present-day Tibetan. Thus, the gene pool of D haplogroup in Qiang minority can be traced back to the ancient Tibetan or the fusion with the historical Tibetan population.

### 4.3. Migration direction and model

The influx of large populations of various haplogroups can rapidly change the genetic landscape of the input site during the long history. Alternatively, relatively large number of Y-STR haplotype data from modern contemporary population have distinct advantage in studying migration route and direction. When we evaluated the gene flow between adjacent Qiangic populations, the bidirectional migration was proved to be the most frequent model except the movements from Wenchuan to Li County. Therefore, gene flow from Wenchuan is vital to the formation of the paternal genetic structure of Li Qiang. Li Qiang showed its particularity once again. The unidirectional migration may attribute to geographical location. Li County is neither along the Mao-Wen ancient route (Qianjiang River Valley) nor along the Min River Valley, but continues to extend west in the valley area (Zaagunao River) with Wenchuan as the node. Beyond this, the migrations between Beichuan-Wenchuan or Beichuan-Li were geographically impossible to cross Mao County, whether forward or backward migration. The results in continuous migration pattern (level two) had been shown to be the predominant model between all the investigated non-adjacent pairs. Li splits off Beichuan, and Mao simultaneously splits off Beichuan and Li. Therefore, the Qiang’s complex movements along Tibetan-Yi corridor had been shown.

In conclusion, the paternal genetic landscapes of Qiang subgroups were uncovered by 37 Y-STR loci. The Y-chromosomal haplogroups from plenty of present-day populations and the archaeological sites provided significant clues to comprehend the origin for present-day Qiang people as well as the migration direction and model. This study will provide valuable clues or information for anthropologists, archaeologists, ethnologists and forensic scientists. However, there are still some limitations in this study. Given the insufficient sample size of Heishui Tibetan, the evaluation for migration along Min River valley is disabled and the relevant LDA results will also become unpersuasive. Meanwhile, Qiang subjects from He-Huang region are still scarce in this study. This Qiangic sub-branch could give a support for Qiang’s origin and long-distance migration history. It is still a large challenge for archaeologists and historians that the archaeological sites of interest are often limited by a very small sample size. With the excavation of more historical remains and ancient human remains, especially the sequencing of ancient DNA from Longshan and YangShao sites, the southwestward migration history of the Q1a1a1 and O2 haplogroup Qiangic populations from WLR region to the Central Plains and eventually to the middle section of Tibetan-Yi corridor is expected to be fully revealed. In addition, although the kNN predictor here has an overall accuracy of up to 96%, the inference of the main haplogroup alone is not sufficient for further historical, genealogical and forensic research. In the future, we are expected to build a more powerful haplogroup prediction tool for plenty of sub-clades of Y-chromosomal haplogroups, and more relevant ethnic groups will participate.

## Data Availability

The datasets supporting this article have been uploaded as part of the electronic supplementary material [45].

## References

[1] Wang CC, Wang LX, Shrestha R, Zhang M, Huang XY, Hu K, Jin L, Li H. 2014 Genetic structure of Qiangic populations residing in the western Sichuan corridor. PLoS One **9**, e103772. (10.1371/journal.pone.0103772) 25090432 PMC4121179

[2] Fan G. 2021 Forensic and phylogenetic analyses of populations in the Tibetan-Yi corridor using 41 Y-STRs. Int. J. Leg. Med. **135**, 783–785. (10.1007/s00414-020-02453-3) 33141282

[3] Matisoff JA. 1991 Sino-Tibetan linguistics: present state and future prospects. Annu. Rev. Anthropol. **20**, 469–504. (10.1146/annurev.an.20.100191.002345)

[4] Cavalli-Sforza L, Menozzi P, Piazza A. 1996 The history and geography of human genes. Princeton, NJ: Princeton University Press.

[5] Deng W *et al*. 2004 Evolution and migration history of the Chinese population inferred from Chinese Y-chromosome evidence. J. Hum. Genet. **49**, 339–348. (10.1007/s10038-004-0154-3) 15173934

[6] Su B *et al*. 2000 Y chromosome haplotypes reveal prehistorical migrations to the Himalayas. Hum. Genet. **107**, 582–590. (10.1007/s004390000406) 11153912

[7] Kang L *et al*. 2012 Y‐chromosome O3 haplogroup diversity in Sino‐Tibetan populations reveals two migration routes into the eastern Himalayas. Ann. Hum. Genet. **76**, 92–99. (10.1111/j.1469-1809.2011.00690.x) 22111786

[8] Xue Y *et al*. 2009 Human Y chromosome base-substitution mutation rate measured by direct sequencing in a deep-rooting pedigree. Curr. Biol. **19**, 1453–1457. (10.1016/j.cub.2009.07.032) 19716302 PMC2748900

[9] Calafell F, Larmuseau MHD. 2017 The Y chromosome as the most popular marker in genetic genealogy benefits interdisciplinary research. Hum. Genet. **136**, 559–573. (10.1007/s00439-016-1740-0) 27817057

[10] Xu H *et al*. 2015 Inferring population structure and demographic history using Y-STR data from worldwide populations. Mol. Genet. Genom. **290**, 141–150. (10.1007/s00438-014-0903-8) 25159112

[11] Fan GY. 2022 Assessing the factors influencing the performance of machine learning for classifying haplogroups from Y-STR haplotypes. Forensic Sci. Int. Genet. **340**, 111466. (10.1016/j.forsciint.2022.111466) 36150277

[12] Yin C *et al*. 2022 Improving the regional Y-STR haplotype resolution utilizing haplogroup-determining Y-SNPs and the application of machine learning in Y-SNP haplogroup prediction in a forensic Y-STR database: a pilot study on male Chinese Yunnan Zhaoyang Han population. Forensic Sci. Int. Genet. **57**, 102659. (10.1016/j.fsigen.2021.102659) 35007855

[13] Petrejčíková E, Čarnogurská J, Hronská D, Bernasovská J, Boroňová I, Gabriková D, Bôžiková A, Mačeková S. 2014 Y-SNP analysis versus Y-haplogroup predictor in the Slovak population. Anthropol. Anz. **71**, 275–285. (10.1127/0003-5548/2014/0368) 25065120

[14] Kayser M *et al*. 2001 An extensive analysis of Y-chromosomal microsatellite haplotypes in globally dispersed human populations. Am. J. Hum. Genet. **68**, 990–1018. (10.1086/319510) 11254455 PMC1275652

[15] Jobling MA, Heyer E, Dieltjes P, de Knijff P. 1999 Y-chromosome-specific microsatellite mutation rates re-examined using a minisatellite, MSY1. Hum. Mol. Genet. **8**, 2117–2120. (10.1093/hmg/8.11.2117) 10484782

[16] Carlson RV, Boyd KM, Webb DJ. 2004 The revision of the Declaration of Helsinki: past, present and future. Br. J. Clin. Pharmacol. **57**, 695–713. (10.1111/j.1365-2125.2004.02103.x) 15151515 PMC1884510

[17] Luo L *et al*. 2021 Forensic characteristics and population construction of two major minorities from southwest China revealed by a novel 37 Y-STR loci system. R. Soc. Open Sci. **8**, 210447. (10.1098/rsos.210447) 34350019 PMC8316789

[18] Nei M. 1987 Molecular evolutionary genetics. New York, NY: Columbia University Press.

[19] Reynolds J, Weir BS, Cockerham CC. 1983 Estimation of the coancestry coefficient: basis for a short-term genetic distance. Genetics **105**, 767–779. (10.1093/genetics/105.3.767) 17246175 PMC1202185

[20] Venables W, Ripley B. 2002 Modern applied statistics with S, 4th edn. New York, NY: Springer.

[21] Altman NS. 1992 An introduction to kernel and nearest-neighbor nonparametric regression. Am. Stat. **46**, 175–185. (10.1080/00031305.1992.10475879)

[22] Song M, Zhao C, Wang Z, Hou Y. 2019 Applying machine learning algorithms to a real forensic case to predict Y-SNP haplogroup based on Y-STR haplotype. Forensic Sci. Int. Genet. Suppl. Ser. **7**, 637–638. (10.1016/j.fsigss.2019.10.120)

[23] Song M *et al*. 2022 Paternal genetic structure of the Qiang ethnic group in China revealed by high-resolution Y-chromosome STRs and SNPs. Forensic Sci. Int. Genet. **61**, 102774. (10.1016/j.fsigen.2022.102774) 36156385

[24] Fan GY, Jiang DZ, Jiang YH, Song W, He YY, Wuo NA. 2023 Phylogenetic analyses of 41 Y-STRs and machine learning-based haplogroup prediction in the Qingdao Han population from Shandong province, Eastern China. Ann. Hum. Biol. **50**, 35–41. (10.1080/03014460.2023.2168057) 36636009

[25] Bandelt HJ, Forster P, Rohl A. 1999 Median-joining networks for inferring intraspecific phylogenies. Mol. Biol. Evol. **16**, 37–48. (10.1093/oxfordjournals.molbev.a026036) 10331250

[26] Young FW. 1970 Nonmetric multidimensional scaling: recovery of metric information. Psychometrika **35**, 455–473. (10.1007/bf02291820)

[27] Hansen J. 2005 Using SPSS for Windows and Macintosh: analyzing and understanding data. Am. Stat. **59**, 113. (10.1198/tas.2005.s139)

[28] Reich D, Price AL, Patterson N. 2008 Principal component analysis of genetic data. Nat. Genet. **40**, 491–492. (10.1038/ng0508-491) 18443580

[29] Moberly JG, Bernards MT, Waynant KV. 2018 Key features and updates for Origin 2018. J. Cheminformatics **10**, 5. (10.1186/s13321-018-0259-x) PMC5807254 29427195

[30] Beerli P, Mashayekhi S, Sadeghi M, Khodaei M, Shaw K. 2019 Population genetic inference with MIGRATE. Curr. Protoc. Bioinform. **68**, e87. (10.1002/cpbi.87) PMC9286049 31756024

[31] Beerli P, Palczewski M. 2010 Unified framework to evaluate panmixia and migration direction among multiple sampling locations. Genetics **185**, 313–326. (10.1534/genetics.109.112532) 20176979 PMC2870966

[32] Girod C, Vitalis R, Leblois R, Fréville H. 2011 Inferring population decline and expansion from microsatellite data: a simulation-based evaluation of the Msvar method. Genetics **188**, 165–179. (10.1534/genetics.110.121764) 21385729 PMC3120153

[33] Lazim H, Almohammed EK, Hadi S, Smith J. 2020 Population genetic diversity in an Iraqi population and gene flow across the Arabian Peninsula. Sci. Rep. **10**, 15289. (10.1038/s41598-020-72283-1) 32943725 PMC7499422

[34] Fan GY, Song DL, Jin HY, Zheng XK. 2021 Gene flow and phylogenetic analyses of paternal lineages in the Yi-Luo valley using Y-STR genetic markers. Ann. Hum. Biol. **48**, 627–634. (10.1080/03014460.2021.2017480) 35072569

[35] Li J, Zeng W, Zhang Y, Ko AMS, Li C, Zhu H, Fu Q, Zhou H. 2017 Ancient DNA reveals genetic connections between early Di-Qiang and Han Chinese. BMC Evol. Biol. **17**, 239. (10.1186/s12862-017-1082-0) 29202706 PMC5716020

[36] Zhao Y, Li H, Li S, Yu C, Gao S, Xu Z, Jin L, Zhu H, Zhou H. 2011 Ancient DNA evidence supports the contribution of Di‐Qiang people to the Han Chinese gene pool. Am. J. Phys. Anthropol. **144**, 258–268. (10.1002/ajpa.21399) 20872743

[37] Ning C *et al*. 2020 Ancient genomes from northern China suggest links between subsistence changes and human migration. Nat. Commun. **11**, 2700. (10.1038/s41467-020-16557-2) 32483115 PMC7264253

[38] Damgaard P de B *et al*. 2018 137 ancient human genomes from across the Eurasian steppes. Nature **557**, 369–374. (10.1038/s41586-018-0094-2) 29743675

[39] Sikora M *et al*. 2019 The population history of northeastern Siberia since the Pleistocene. Nature **570**, 182–188. (10.1038/s41586-019-1279-z) 31168093 PMC7617447

[40] Wells RS *et al*. 2001 The Eurasian heartland: a continental perspective on Y-chromosome diversity. Proc. Natl Acad. Sci. USA **98**, 10244–10249. (10.1073/pnas.171305098) 11526236 PMC56946

[41] Liu L. 2007 The Chinese Neolithic: trajectories to early states. Cambridge, UK: Cambridge University Press.

[42] Wang M. 1992 The Ch’iang of ancient China through the Han dynasty: ecological frontiers and ethnic boundaries. Cambridge, MA: Harvard University.

[43] Shi S. 2018 Ethnic flows in the Tibetan-Yi corridor throughout history. Int. J. Anthropol. Ethnol. **2**, 2. (10.1186/s41257-018-0009-z)

[44] Duan L, Gong Q. 2007 The origin of Di-Qiang ethnic group in southwest China. J. Guangxi Univ. Natl. **29**, 44–48. (10.3969/j.issn.1673-8179.2007.04.010)

[45] Fan GY, Zhu Y, Wang EN, Ju TG. 2025 Supplementary material from: Investigating the paternal genetic structure and migration history of Chinese Qiang minority by Y-chromosome STRs. FigShare. (10.6084/m9.figshare.c.7823318) PMC12092108 40400520

